# Safety of Normothermic Cardiopulmonary Bypass in Pediatric Cardiac Surgery: A System Review and Meta-Analysis

**DOI:** 10.3389/fped.2021.757551

**Published:** 2021-12-14

**Authors:** Tao Xiong, Lei Pu, Yuan-Feng Ma, Yun-Long Zhu, Xu Cui, Hua Li, Xu Zhan, Ya-Xiong Li

**Affiliations:** ^1^Department of Cardiac Surgery, Kunming Yan'an Hospital, Affiliated Hospital of Kunming Medical University, Kunming, China; ^2^Cardiovascular Surgery, Institution of Yunnan, Kunming, China

**Keywords:** normothermia, congenital heart surgery, cardiopulmonary bypass, meta-analysis, cardiac surgery

## Abstract

**Objectives:** Hypothermic cardiopulmonary bypass (HCPB) has been used successfully in cardiac surgery for more than half a century, although adverse effects have been reported with its use. Many studies on temperature management during CPB published to date have shown that normothermic CPB (NCPB) provides more benefits to children undergoing cardiac surgery. The present meta-analysis investigated the effect of NCPB on clinical outcomes based on results of randomized controlled trials and observational studies on pediatric cardiac surgery.

**Methods:** Databases such as PubMed, EMBASE, Cochrane Central Register of Controlled Trials, and Clinical Trials.gov were searched from inception to May 2021 to identify relevant studies published in English.

**Results:** The present meta-analysis included 13 studies characterizing a total of 837 pediatric patients. The random effects model exhibited that the NCPB group had reduced revision for postoperative bleeding [odds ratio (OR): 0.11; 95% confidence interval (CI): 0.01–0.89; *I*^2^ = 0%, *P* = 0.04], serum lactate 2–4 h after CPB (mean difference: −0.60; 95% CI: −1.09 to −0.11; *I*^2^ = 82%, *P* = 0.02), serum creatinemia 24 h after CPB (mean difference: −2.73; 95% CI: −5.06 to −0.39; *I*^2^ = 83%, *P* = 0.02), serum creatinemia 48 h after CPB (mean difference: −2.08; 95% CI: −2.78 to −1.39; *I*^2^ = 0%, *P* < 0.05), CPB time (mean difference: −19.10, 95% CI: −32.03 to −6.18; *I*^2^ = 96%, *P* = 0.04), and major adverse events (OR: 0.37; 95% CI: 0.15–0.93; *Z* = 2.12, *P* = 0.03) after simple congenital surgery compared with the HCPB group.

**Conclusion:** NCPB is as safe as HCPB in pediatric congenital heart surgery. Moreover, NCPB provides more advantages than HCPB in simple congenital heart surgery.

## Introduction

Hypothermic cardiopulmonary bypass (HCPB), alone or associated with deep hypothermic circulatory arrest, is widely used in pediatric cardiac surgery (1). Most operations are performed at the temperature between 28 and 30°C (2). The rationale for body cooling is to protect the brain, kidneys, and heart from ischaemic injury by reducing the metabolic rate and decreasing oxygen consumption (3). However, clinical research provides increasing evidence for the harmful effects of hypothermia such as interference with oxygen and glucose uptake in the brain of pediatric patients (4). These adverse affects result in a transient period of increased intracranial pressure and a long period of neurophysiologic dysfunction, thrombocytic and coagulation dysfunction (5). Recently, normothermic cardiopulmonary bypass (NCPB) has become increasingly popular in adult cardiac surgery. Studies have reported the absence of shivering, haemodynamic stability, mimimal use of inotropes, and early extubation when the body of patients was not cooled (6–8). These observations led researchers to investigate the effect of systemic normal temperature perfusion on organ function during pediatric heart surgery. In infants and children undergoing cardiac surgery, NCPB has been shown to reduce oxidative stress and result in similar myocardial reperfusion, renal injury, and inflammatory response as HCPB (9, 10), provide a more physiologically preserved balance of ATP or energy supply and demand, and improve late neurodevelopmental outcomes (11). In a study conducted by the Siyami Ersek Thoracic and Cardiovascular Surgery Center, the NCPB group exhibited higher values of whole-body oxygen delivery (DO_2_), consumed oxygen (VO_2_), and whole-body oxygen extraction fraction than the HCPB groups at 20 and 60 min after aortic cross clamp, end of CPB, and 2 h after CPB (*P* < 0.0001), which indicated that the use of NCPB provides better gastric mucosal oxygenation than HCPB in neonates and infants undergoing congenital heart surgery with CPB procedures (12). However, intraoperative experience exhibited that NCPB is more frequently associated with cardioplegia, causing increased duration of aortic cross-clamping (12).

Thus, the present review attempted to evaluate the safety of NCPB compared with that of HCPB through a meta-analysis of relevant studies.

## Methods

The present study was performed in accordance with the Preferred Reporting Items for Systematic Reviews and Meta-analyses (PRISMA) guidelines.

### Search Strategies

Electronic databases, namely PubMed, EMBASE, Cochrane Central Register of Controlled Trials (CENTRAL), ClinicalTrials.gov of systematic reviews, and Lei Pu were searched for original publications. Keywords in the title or text as well as MeSH terms related to heart surgery, namely “paediatric,” “child,” “infant,” “cardiac surgery,” “thoracic surgery,” “cardiothoracic surgery,” “heart surgery,” “congenital heart surgery,” “pediatric cardiology,” “cardiopulmonary bypass,” “CPB,” “normal temperature,” “normal thermic” “normothermic,” and “normothermia,” were used as search terms (Supplementary Material 1). The searches were limited to studies published in English language.

### Selection Criteria

Randomized and observational studies were included in the initial search. Studies published in English language, comparing normothermia (systemic perfusion or core temperature ≥34°C) with hypothermia (<32°C) as an intraoperative temperature strategy, were included. Studies using different cardioplegia temperature but same core perfusion temperature were excluded; studies on off-pump cardiac surgery or circulatory arrest; those with no control group; those conducted on animals and with an experimental design; and those conducted on adults (≥18 years) were excluded.

The criteria were predefined, and the search process was performed by two investigators (M.Y.F. and Z.Y.L.). Discrepancies were resolved through discussions.

### Data Extraction and Quality Assessment

Data extraction and presentation followed the recommendations of the PRISMA statement (13). Data regarding study characteristics, patient characteristics, and outcomes were extracted. The methodological quality of the included studies was based on the recommendation of the PRISMA statement (13) and the Cochrane Handbook for Systematic Reviews of Interventions (14). All authors independently assessed the quality of selected studies using the preliminary Risk of Bias (RoB) in Non-randomized Studies of Exposures (ROBINS-E) tool (15). This study covers seven aspects, including: (1) bias due to confounding; (2) bias in selecting participants in the study; (3) bias in exposure classification; (4) bias due to departures from intended exposures; (5) bias due to missing data; (6) bias in outcome measurement; (7) bias in the selection of reported results. Each region is characterized by low, moderate, and severe risk of bias. We report the criteria for assessing the risk of bias in Supplementary Table 2. If the evaluator disagrees, we assign a majority approval rate.

### Data Analysis

Statistical analysis was conducted according to the recommendations of the Cochrane Intervention Systematic Evaluation Manual (14). Classification variables were estimated using the Mantel–Haenszel odds ratio (OR) and 2-tailed 95% confidence interval (CI), whereas the continuous variables were analyzed using a weighted mean difference (WMD). *I*^2^ statistics evaluated by the Q test were used to quantify the degree of heterogeneity between studies. Given inherent differences in study design, the OR-weighted average difference estimates were calculated for all comparisons by using a random effects model.

Publication bias was assessed using the Begg adjusted rank correlation test and Egger regression asymmetry test (14). To explore the influence of covariates on the CPB time effect, a random effects metaregression analysis was performed, in which the logarithm of the WMD of the major outcomes was regressed according to the included baseline characteristics. All *P*-values were 2-tailed, and a *P*-value of <0.05 was considered statistically significant. All statistical analyses were performed using RevMan version 5.3 (The Cochrane Collaboration) and Stata version 16.0 (Statacorp).

## Results

### Baseline Characteristics

**Figure 1:**
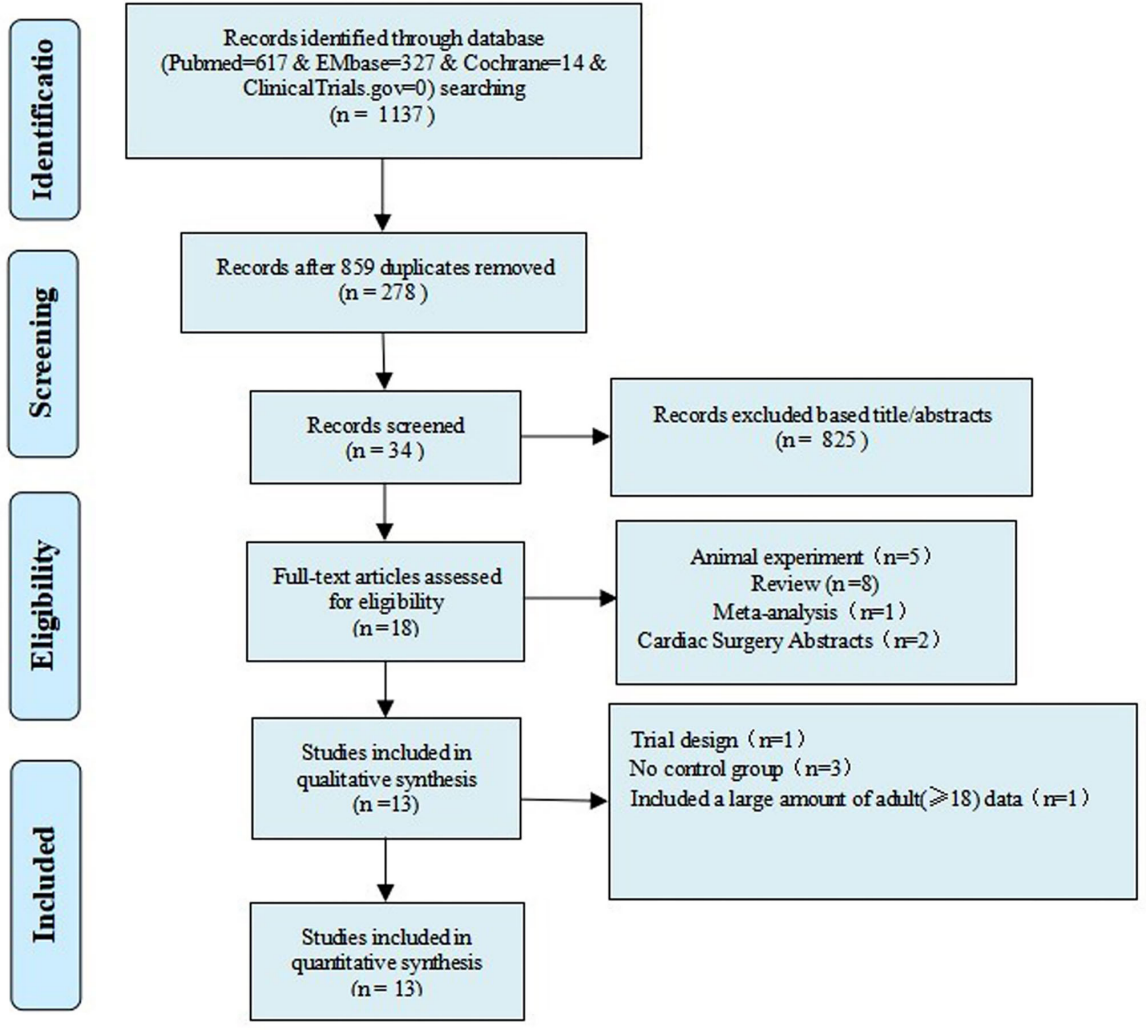
Flow chart of the selection process for studies included in the systematic review and meta-analysis.

Supplementary Material 1 illustrates the search strategy. Using the search strategy, 1,137 citations were retrieved, and 278 duplicates were excluded. The remaining 859 articles were screened based on the title, abstract, article type, and language, and 839 articles were excluded. The remaining 20 studies were subjected to a full-text review with predefined inclusion criteria, following which five studies were excluded (Figure 1). The reasons for exclusion are illustrated in Table 1. Caputo et al. published their studies in 2005 and 2011 and reported different results on the same premise of the data included in 2005; their study published in 2005 was conducted from 2002 to 2004 (9) and reported on myocardial injury, oxidative stress, and inflammatory response in pediatric open heart surgery, whereas their study published in 2011 (10) reported on renal injury in pediatric open heart surgery. Because the baseline characteristics of the two studies were the same, both studies were merged as Caputo 2005/2011. Additionally, Caputo et al. published the second phase of the study in 2018 (21) and included the 2005 (first phase) study for a comprehensive analysis. Therefore, in this meta-analysis, only the two-stage data of the study were extracted. The sample included in the study by Karaci et al. (12) was divided into two groups (pulsatile and non-pulsatile CPB) for research, which are represented as studies by Karaci, including Karaci-a 2011 and Karaci-b 2011, respectively. Therefore, 13 unique studies characterizing 837 pediatric patients were selected in our meta-analysis (9–12, 21–29). The baseline characteristics of the included studies are summarized in Table 2, the full statistical characteristics of the included studies are summarized in Supplementary Table 7, and the quality assessment for included trials is presented in Table 3. The outcome data of included studies.

**Table 1:** Reasons for exclusion.

**References**	**Reason for exclusion**
Baos et al. (16)	Study was a trial design
Chowdhury et al. (17)	Study included a large amount of adult (≥18) data
Shamsuddin et al. (18)	Studies had no control group
Durandy and Hulin (19)	Studies had no control group
Padhy et al. (20)	Studies had no control group

**Table 2:** Baseline characteristics of individual studies.

**References**	**Countries**	**Operation period**	**Study design**	**Number of patients**	**Number of male**		**Age/(month)**		**Temperature**	
				**NCPB/HCPB**	**NCPB group**	**HCPB group**	**NCPB group**	**HCPB group**	**NCPB group**	**HCPB group**
Pouard et al. (28)	France	October 2000 to October 2001	PC	40/(20/20)	NR	NR	6.19 ± 1.35	5.45 ± 5.5	35-36°C	23-25°C
Amer et al. (23)	Egypt	October 2017 to February 2019	RCT	40/(20/20)	13	12	13.5 ± 17.9	15.5 ± 18.3	35-37°C	32-35°C
Caputo et al. (9, 10)	United Kingdom	November 2002 to November 2004	RCT	59/(28/31)	9	16	89.02 ± 21.3	59 ± 27.26	35–37°C	28°C
Ly et al. (27)	France	2000 to 2008	RC	110/(40/70)	NR	NR	2.57 ± 6.34	23.28 ± 37.34	≥34°C	≤28°C
Poncelet et al. (11)	Belgium	May 2004 to September 2005	RCT	47/(22/25)	NR	NR	18.83	24.33	36.5°C	32°C
Corno et al. (24)	United States	January 2014 to December 2015	RC	99/(48/51)	NR	NR	7.7 ± 6.1	6.6 ± 6.5	≥35°C	<28°C
Caputo et al. (21)	United Kingdom	2012 to 2014	RCT	141/(70/71)	30	31	2.38 ± 0.99	2.95 ± 1.16	35-36°C	29°C
Hannon et al. (25)	United Kingdom	January 2014 to December 2015	RC	95/(45/50)	25	29	6.97 ± 1.26	6.2 ± 1.57	32–35°C	<28°C
Kim et al. (26)	Korea	January 2001 to December 2010	PC	43/(24/19)	15	11	4.1 ± 2.9	3.3 ± 2.12	34-36°C	26–29°C
Stocker et al. (29)	Australia	March 2003 to July 2005	RCT	54/(27/27)	16	16	7.45 ± 5.86	6.17 ± 5.51	34°C	24°C
Karaci-a et al. (12)	United States	January 19 to March 27, 2011	PC	30/(15/15)	15	15	3.8 ± 2.5	4.4 ± 2.4	35°C	<28°C
Karaci-b et al. (12)	United States	January 19 to March 27, 2011	PC	30/(15/15)	15	15	3.2 ± 2.6	4.2 ± 2.7	35°C	<28°C
Abdul-Khaliq et al. (22)	Germany	NR	PC	49/(20/29)	20	29	20.2 ± 22.7	11 ± 55	36°C	22-35°C
	**Number/major diseases**				**BSA/m** ^2^		**Cross-clamp time, minutes**		**CPB time, minutes**	
	**NCPB group**	**HCPB group**			**NCPB group**	**HCPB group**	**NCPB group**	**HCPB group**	**NCPB group**	**HCPB group**
	20/TGA	20/TGA			NR	NR	79.5 ± 13.1	78.4 ± 10.9	125.7 ± 11.5	128.1 ± 14.1
	20/AVSD	20/AVSD			0.55 ± 0.12	0.63 ± 0.35	72.45 ± 11.97	76.85 ± 10.07	96.40 ± 14.36	103.25 ± 13.92
	20/AVSD + ASD + VSD 0/TOF	26/AVSD + ASD + VSD 0/TOF			0.90 ± 0.12	0.73 ± 0.17	22.43 ± 4.60	33.4 ± 5.36	49.49 ± 6.71	62.23 ± 7.79
	40/IAA	70/CAA			NR	NR	61.7 ± 29.3	87.9 ± 31.2	112.3 ± 64.8	173 ± 71.3
	16/AVSD + ASD + VSD 4/TOF	20/AVSD + ASD + VSD TOF=4			NR	NR	NR	NR	90	94
	22/AVSD + ASD + VSD 9/TOF	23/AVSD + ASD + VSD 11/TOF			NR	NR	58 ± 37	76 ± 37	94 ± 41	116 ± 41
	43/AVSD + ASD + VSD 9/TOF	40/AVSD + ASD + VSD 12/TOF			0.5 ± 0.08	0.51 ± 0.11	60.64 ± 8.65	45.69 ± 11.79	90.36 ± 9.92	74.41 ± 10.95
	NR	NR			NR	NR	NR	NR	NR	NR
	24/VSD	19/VSD	NR			NR	37 ± 9	54 ± 16	59 ± 12	88 ± 25
	14/AVSD + ASD + VSD 8/TOF	16/AVSD + ASD + VSD 6/TOF			NR	NR	75 ± 31	83 ± 31	110.67 ± 19.28	139.11 ± 18.78
	9/AVSD + VSD 3/TOF	6/AVSD + VSD 3/TOF			0.24 ± 0.04	0.25 ± 0.08	61 ± 34	64 ± 45	79 ± 39	81 ± 46
	9/AVSD + VSD 5/TOF	9/AVSD + VSD 4/TOF			0.23 ± 0.05	0.25 ± 0.03	59 ± 29	55 ± 22	68 ± 33	77 ± 28
	15/AVSD + VSD + ASD 5/TOF	13/AVSD + VSD 4/TOF			NR	NR	NR	NR	37 ± 30	102 ± 41

*NCPB, normothermic cardiopulmonary bypass; HCPB, hypothermic cardiopulmonary bypass; TGA, transposition of the great arteries; VSD, ventricular septal defect; ASD, Atrial septal defect; IAA, interruption of aortic arch; CAA, Coarctation aortic arch; AVSD, atrioventricular septal defect; TOF, tetralogy of Fallot; BSA, body surface area; CPB, cardiopulmonary bypass; Kg, kilogram; NR, no reported; PC, prospective cohort;RCT, randomized clinical trial; RC, retrospective controlled; a, non-pulsatile cardiopulmonary bypass; b, pulsatile cardiopulmonary bypass*.

**Table 3:** Quality assessment.

**References**	**Bias due to Confounding**	**Bias in selection of participants into study**	**Bias in classification of interventions**	**Bias due to deviations from intended intervention**	**Bias due to missing bias**	**Bias in measurement of outcomes**	**Bias in selection of the reported result**	**Risk score**
Pouard et al. (28)	Low risk	Low risk	Low risk	Low risk	Low risk	Low risk	Low risk	Low risk
Karaci et al. (12)	Low risk	Low risk	Low risk	The risk is not clear	Low risk	Low risk	The risk is not clear	Low risk
Amer et al. (23)	Low risk	Low risk	Low risk	The risk is not clear	Low risk	Low risk	The risk is not clear	Low risk
Caputo et al. (9)	Low risk	Revere risk	Low risk	Low risk	Low risk	Low risk	Low risk	Low risk
Abdul-Khaliq et al. (22)	Revere risk	Low risk	Low risk	Low risk	Low risk	Low risk	The risk is not clear	Revere risk
Ly et al. (27)	Revere risk	Low risk	Low risk	The risk is not clear	Low risk	Low risk	The risk is not clear	Revere risk
Poncelet et al. (11)	Revere risk	Low risk	Low risk	Revere risk	Revere risk	Low risk	Revere risk	Revere risk
Corno et al. (24)	Revere risk	Low risk	Low risk	Revere risk	Low risk	Low risk	The risk is not clear	Revere risk
Caputo et al. (21)	Low risk	Low risk	Low risk	Low risk	Low risk	Low risk	Low risk	Low risk
Hannon et al. (25)	Low risk	Low risk	Low risk	Low risk	Low risk	Low risk	The risk is not clear	Low risk
Kim et al. (26)	Revere risk	Low risk	Low risk	Low risk	Low risk	Low risk	Low risk	Revere risk
Caputo et al. (10)	Low risk	Low risk	Revere risk	Low risk	Low risk	Low risk	Low risk	Low risk
Stocker et al. (29)	Low risk	Low risk	Low risk	Low risk	Low risk	Low risk	Low risk	Low risk

### Death

**Figure 2:**
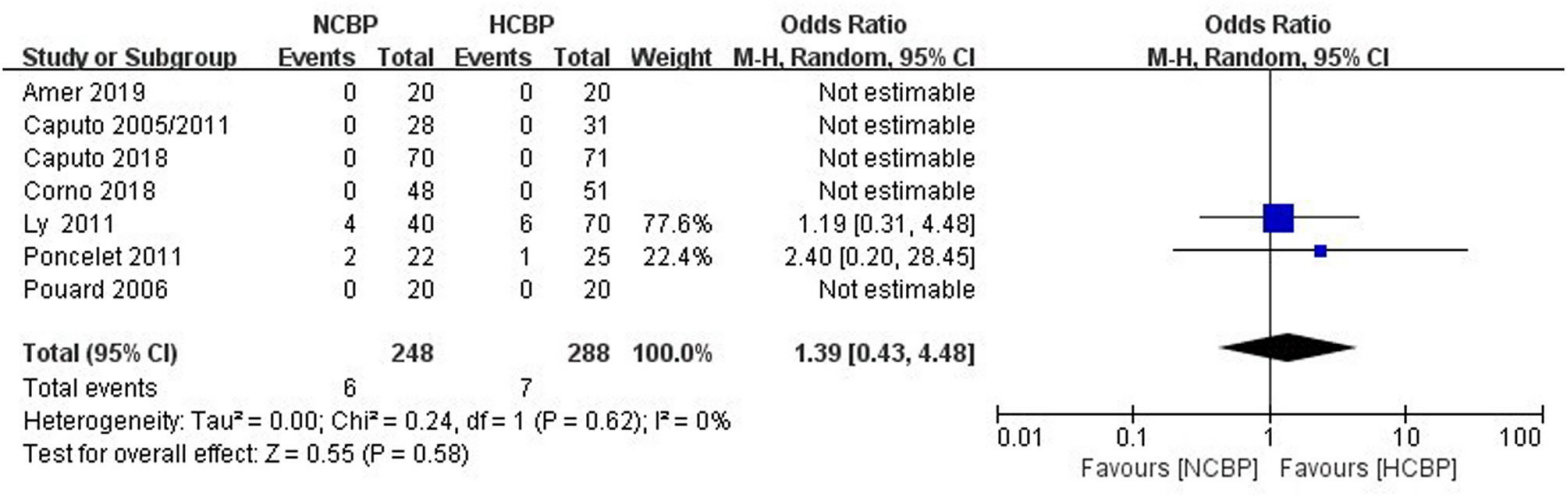
Pooled analysis for the comparison of the risk of death.

The NCPB group with 248 patients exhibited six deaths, whereas the HCPB group with 288 patients exhibited 7 deaths (Figure 2). The pooled analysis of this outcome exhibited a similar death rate between the NCPB and HCPB groups (OR: 1.39; 95% CI: 0.43–4.48; *Z* = 0.55, *P* = 0.58). Among them, all studies are divided into non-RCTs (OR: 2.0; 95% CI: 0.53–7.49; *Z* = 1.03, *P* = 0.30; Supplementary Figure 1) and RCTs (OR: 2.06; 95% CI: 0.18–23.24; *Z* = 0.58, *P* = 0.56; Supplementary Figure 2) subtypes, and the pool analysis shows not enough evidence for a difference between the two.

### Main Adverse Events After Surgery

**Figure 3:**
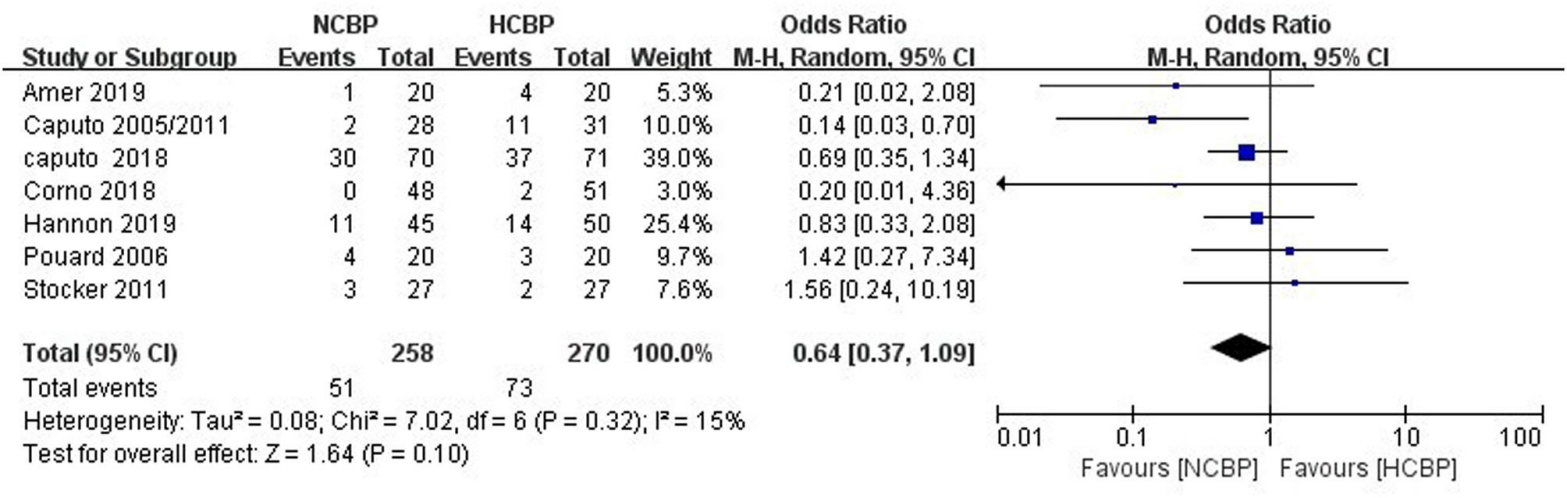
Pooled analysis for the total adverse events.

**Figure 4:**
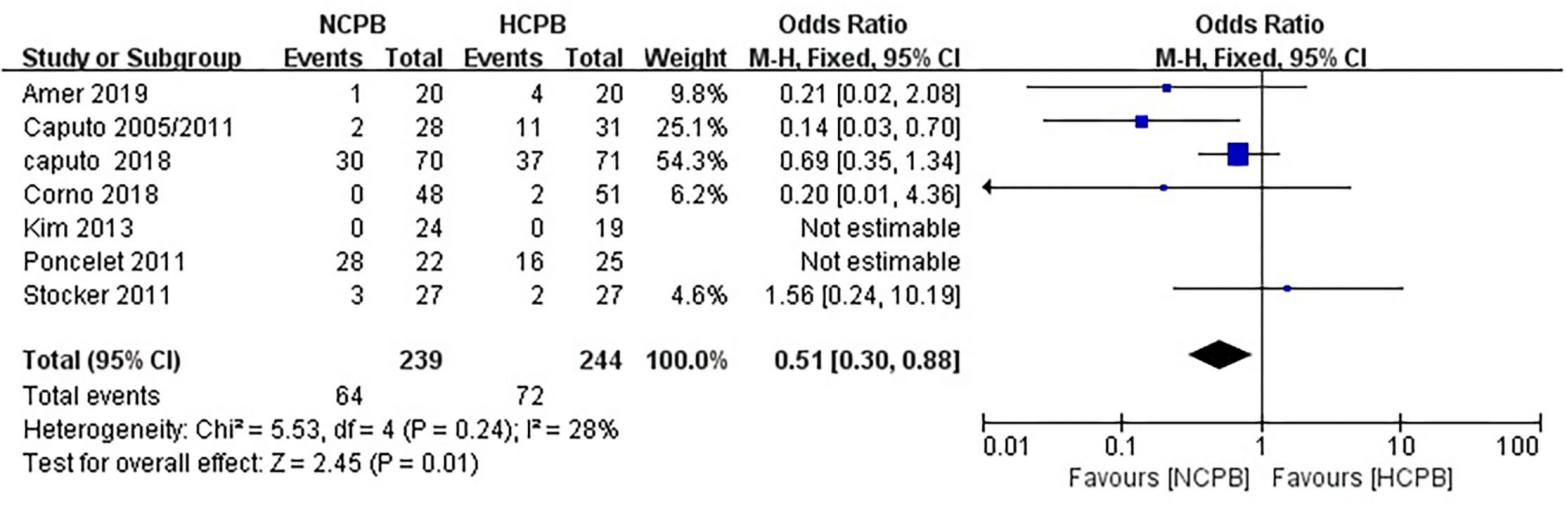
Pooled analysis for major adverse events after simple congenital heart disease surgery.

Because not all studies had reported the same outcome indicators, and only some of the adverse events had been reported in each study (Supplementary Table 1), but still, pool analysis of total adverse events showed no significant difference between NCPB groups and HCPB groups(OR: 0.64; 95% CI: 0.37–1.09; *Z* = 1.64, *P* = 0.10; Figure 3). The main adverse events of children were analyzed through subgroup analysis based on the complexity of surgery. First, the study by Hannon et al. (24) was excluded because it did not mention the type of congenital heart disease; secondly, the study by Pouard et al. (28) was excluded because the included children were all transposition of the great arteries (TGAs); finally, the study by Ly et al. (29) was excluded because the included children were interruption of aortic arch (IAA) and coarctation aortic arch (CAA). Pooled analysis of the main adverse events of the remaining studies indicated that the incidence of main adverse events associated with NCPB was lower than that associated with HCPB after simple congenital heart disease surgery, and the difference was statistically significant (OR: 0.51; 95% CI: 0.30–0.88; *Z* = 2.45, *P* = 0.01; Figure 4). Addition, the meta-analysis of specific adverse events was presented in the subgroup analysis, as shown in Supplementary Table 2. Among them, all studies are divided into non-RCT (OR: 0.86; 95% CI: 0.39–1.86; *Z* = 0.39, *P* = 0.69; Supplementary Figure 3) and RCT (OR: 0.48 95% CI: 0.19–1.24; *Z* = 1.51, *P* = 0.13; Supplementary Figure 4) subtypes, and the pool analysis shows not enough evidence for a difference between the two.

The pooled analysis of three studies provided data on the outcome of delayed chest closure (OR: 0.7; 95% CI: 0.32–1.52; *Z* = 0.9, *P* = 0.37; Supplementary Figure 5). Similarly, the pooled analysis of five studies provided data on the outcome of arrhythmia (OR: 0.84; 95% CI: 0.42–1.68; *Z* = 0.48, *P* = 0.63; Supplementary Figure 6), that of three studies provided data on the outcome of left ventricular failure (OR: 0.91; 95% CI: 0.46–1.81; *Z* = 0.26, *P* = 0.79; Supplementary Figure 7), that of three studies provided data on the outcome of pulmonary complications (OR: 0.78; 95% CI: 0.33–1.87; *Z* = 0.55, *P* = 0.58; Supplementary Figure 8), that of five studies provided data on the outcome of neurological complications (OR: 1.14; 95% CI: 0.07–19.42; *Z* = 0.09, *P* = 0.93; Supplementary Figure 9), that of four studies provided data on the outcome of renal complications (OR: 1.01; 95% CI: 0.06–16.54; *Z* = 0.01, *P* = 0.99; Supplementary Figure 10), that of four studies provided data on the outcome of blood loss (mean difference: −4.27; 95% CI: −16.87 to 8.32; *Z* = 0.66, *P* = 0.51; Supplementary Figure 11), that of four studies provided data on the outcome of reoperation (OR: 0.56; 95% CI: 0.26–1.21; *Z* = 1.48, *P* = 0.14; Supplementary Figure 12), and that of three studies provided data on the outcome of infective complications (OR: 1.41; 95% CI: 0.71–2.80; *Z* = 0.97, *P* = 0.33; Supplementary Figure 13). These results did not provide sufficient evidence to confirm the difference between the NCPB and HCPB groups.

The data on the outcome of revision for bleeding had been reported in two studies (OR: 0.11; 95% CI: 0.01–0.89; *I*^2^ = 0%, *P* = 0.04; Supplementary Figure 14). The results exhibited that the use of NCPB could reduce events of revision for bleeding compared with HCPB.

### Serum Lactate and Creatinemia Levels

The present study analyzed changes in the serum lactate and creatinemia levels at the blood biochemical level to further study the effects of NCPB on kidney functions and inflammatory response of body tissues.

By analyzing the results of related studies (Supplementary Table 3), the serum lactate levels were divided into 5 time ranges (Supplementary Table 4). The pooled analysis was performed to determine the baseline level of serum lactate (before CPB) (mean difference: 0.04; 95% CI: −0.09 to 0.17; *Z* = 0.58, *P* = 0.56; six trials; Supplementary Figure 15), and those at the end of CPB (mean difference: 0.10; 95% CI: −0.41 to 0.60; *Z* = 0.38, *P* = 0.70; seven trials; Supplementary Figure 16), 2–4 h after CPB (mean difference: −0.60; 95% CI: −1.09 to −0.11; *I*^2^ = 82%, *P* = 0.02; six trials; Supplementary Figure 17), 6–12 h after CPB (mean difference: −0.12; 95% CI: −0.42 to 0.19; *Z* = 0.75, *P* = 0.45, three trials; Supplementary Figure 18), and 12–48 h after CPB (mean difference: −0.46; 95% CI: −1.02 to 0.10; *Z* = 1.62, *P* = 0.11; five trials; Supplementary Figure 19). Among these, the pooled results for the 2–4 h time range exhibited that the use of NCPB can reduce serum lactate levels 1–2 days after CPB. Outcomes for the remaining time periods did not provide sufficient evidence to confirm the difference between the NCPB and HCPB groups.

The studies reporting the analysis of the serum creatinemia level exhibited 5 time points (Supplementary Table 5). The serum creatinemia level at baseline (mean difference: −0.20; 95% CI: −1.23–0.83; *Z* = 0.37; *P* = 0.71; two trials; Supplementary Figure 20) and after CPB (mean difference: −0.62; 95% CI: −4.68 to 3.43; *Z* = 0.30; *P* = 0.76; two trials; Supplementary Figure 21) exhibited no difference between the NCPB and HCPB groups. The level of serum creatinemia 24 h after CPB (mean difference: −2.73; 95% CI: −5.06 to −0.39; *I*^2^ = 83%, *P* = 0.02; three trials; Supplementary Figure 22) and 48 h after CPB (mean difference: −2.08; 95% CI: −2.78 to −1.39; *I*^2^ = 0%, *P* < 0.05; three trials; Supplementary Figure 23) indicated that NCPB reduced serum creatinemia levels compared with HCPB. The serum creatinemia level 72 h after CPB (mean difference: −0.93; 95% CI: −4.35 to 2.48; *Z* = 0.54; *P* = 0.59; three trials; Supplementary Figure 24) did not provide sufficient evidence to confirm the difference between the NCPB and HCPB groups.

### Intraoperative and Hospitalization Data

The outcome data of CPB time had been reported in 13 studies (Supplementary Tables 6, 7). The pooled analysis using a random effects model exhibited that NCPB is associated with a lower CPB time than HCPB (mean difference: −19.10, 95% CI: −32.03 to −6.18; *I*^2^ = 96%, *P* = 0.04; Supplementary Figure 25). The outcome data of aortic clamping time had been reported in 11 studies; the pooled analysis of this outcome exhibited no significant difference between the NCPB and HCPB groups (mean difference: −6.72, 95% CI: −16.46 to 3.02; *Z* = 1.35, *P* = 0.18; Supplementary Figure 26). The outcome data of mechanical ventilation time had been reported in six studies; the pooled analysis of this outcome exhibited no significant difference between the NCPB and HCPB groups (mean difference: −5.94, 95% CI: −22.78 to 10.89; *Z* = 0.69, *P* = 0.49; Supplementary Figure 27). The outcome data of hospital stay had been reported in 3 studies; the pooled analysis of this outcome did not provide sufficient evidence to confirm the difference between the NCPB and HCPB groups (mean difference: −0.58, 95% CI: −1.24 to 0.09; *Z* = 1.7; *P* = 0.09; Supplementary Figure 28). The outcome data of intensive care unit (ICU) hospital stay had been reported in 5 studies. The pooled analysis of this outcome did not provide adequate evidence to confirm the difference between the NCPB and HCPB groups (mean difference: −0.35, 95% CI: −0.72 to 0.02; *Z* = 1.84; *P* = 0.07; Supplementary Figure 29).

### Metaregression Analysis

The regression analysis exhibited that CPB time and known baseline variables such as sample size, region, operation period, study type, major disease, and temperature were not statistically significant (Table 4).

**Table 4:** Metaregression analyses.

**Covariates**	**No. of observations**	***t***	***P*-value**	**Significance with the logarithm MD**
Operation period	11	0.63	0.565	No
Countries	11	−1.76	0.154	No
Sample	11	−1.56	0.193	No
Study design	11	−1.31	0.261	No
Major diseases	11	−2.12	0.101	No
Temperature	11	−0.34	0.749	No

*MD, mean difference*.

### Sensitivity Analysis

NCPB exhibited lower CPB time, events of revision for postoperative bleeding, serum lactate levels 2–4 h after CPB, and serum creatinemia levels 24 h and 48 h after CPB than HCPB. However, only a few studies had focused on revision for postoperative bleeding and serum lactate and creatinemia levels. Therefore, in the present study, the sensitivity analysis was not feasible and was conducted only on CPB time. Upon exclusion of any of the studies, the mean difference changed from −14.59 to −21.01 (Supplementary Figure 30), with no significant change. The considerable heterogeneity (*I*^2^ > 75%) may be due to the clinical heterogeneity such as the technique used by the surgeon or the complexity of congenital heart disease.

### Publication Bias

The present study explored the publication bias of the influence of NCPB on the CPB time. Results of the Begg (*P* = 0.755) and Egger (*P* = 0.062) tests indicated no publication bias.

## Discussion

The practice of systemic cooling differs greatly among congenital cardiac surgeons, and a large proportion of surgeons still prefer to cool the body of the child to 26–30°C (30). However, the normal temperature CPB perfusion strategy has been applied only in research on adult cardiac surgery, and thus, it cannot be directly applied to children.

Melrose et al. proposed the concept of normal temperature myocardial protection in 1955 (31). The advantages of blood cardioplegia over crystalloid cardioplegia were experimentally proven in adults (24) and were clinically proven later in the pediatric population (32). The present study is particularly crucial in pediatric heart surgery because any new surgical method may affect patient safety and durability of surgical repair.

The present meta-analysis demonstrated that the CPB time associated with NCPB is lower than that associated with HCPB among children undergoing cardiac surgery; however, the CI range after pooled analysis varied greatly, which indicated the low reliability of the results. Besides our clinical experience suggests that the CPB time is prolonged while using hypothermia compared with that under normothermia because a fair amount of time is spent in rewarming the patient. In addition, the incidence of events of revision for postoperative bleeding was lower with NCPB than with HCPB among children undergoing cardiac surgery. However, only two studies included in the meta-analysis had considered the revision for bleeding as an outcome variable. Causal association with temperature on CPB could be inferred because of the presence of several confounding variables for revision for bleeding that have not been considered (or Could Not be considered because of the small numbers studies.) in the analysis. Additionally, the serum lactate levels 2–4 h after CPB and serum creatinemia levels 24 and 48 h after CPB were lower with NCPB than with HCPB, indicating that the use of NCPB can improve body inflammation and kidney function over a period of time after surgery. These results were not reported in the previous meta-analysis (33). However, the outcomes for lactate levels may be biased by the treatment used for children (e.g., blood products, Ringer lactate, and cardioplegia), Therefore, this result should be cautiously treated. Simultaneously, a pooled analysis of the time endpoints of serum lactate and creatinine levels exhibited no difference between the two groups (*P* > 0.05), indicating that the difference in the body inflammatory response and renal function between NCPB and HCPB is similar after a long period of recovery following surgery. This result is similar to that of the previous meta-analysis (33). And the above results are similar to the results of several high-quality studies and further prove the safety of NCBP. For example, Caputo et al. (9) discovered that NCPB reduces oxidative stress to a higher degree than HCPB by causing release of troponin and inflammation markers and finally exhibits similar myocardial reperfusion injury and whole body inflammatory response as HCPB. Additionally, they found that aortic clamping and CPB times were significantly longer in the HCPB group than in the NCPB group (*P* = 0.0096 and *P* = 0.030, respectively). Caputo et al. (10) hypothesized that body temperature perfusion is an effective physiological method to maintain the functional integrity of major organ systems. Research has shown that normal temperature CPB and HCPB exhibit similar renal damage in children undergoing cardiac surgery; however, more high-quality studies are required to validate these findings.

The present meta-analysis included new research and further analysis on the occurrence of the main adverse events after surgery (excluding the events of revision for postoperative bleeding), death, aortic clamping time, postoperative mechanical ventilation time, hospitalization, and ICU hospital stay. The pooled analysis indicated no difference between the NCPB and HCPB groups in terms of these variables, suggesting that NCPB and HCPB exhibit similar safety in pediatric heart surgery. Moreover, this study divided all the studies into two subgroups, namely RCT and non-RCT, and performed pooled analysis of death events and major adverse events, although evidence to confirm the difference between the two groups was not sufficient, considering the small number of studies reporting adverse events and the suboptimal quality of the analysis. However, in this study, it was found that the incidence of major adverse events in the NCBP group after simple congenital heart surgery was lower than that in the HCBP group, which can provide favorable evidence for CPB temperature management decisions for simple congenital heart surgery. In addition, the aforementioned analysis was performed without the original data such as age and weight, and thus, the subgroup analysis such as for low-weight and normal-weight infants and children, could not be performed, which resulted in a high clinical heterogeneity. Therefore, the results of pooled analysis should be viewed dialectically.

Presently, several high-quality prospective randomized controlled trial (RCT) studies have reported the benefits of NCPB in pediatric cardiac surgery. Abdul-Khaliq et al. (22) demonstrated that newborns undergoing deep hypothermia CPB may experience a transient increase in cerebrovascular resistance during early stages, making them vulnerable to postoperative neurological complications. Therefore, NCPB may be more advantageous than HCPB under certain conditions. Simultaneously, Karaci et al. (12) used a gastrotonometer to detect visceral hypoxia for determining the systemic oxygen consumption in children with congenital heart surgery under CPB. According to the measured value of gastric pressure, the influence of NCPB on whole-body oxygen delivery (DO2) and consumption (VO2), and oxygen (O_2_) intake is better than HCPB. Poncelet et al. (11) conducted research on postoperative neurological complications in 18 children, with an average of 4 years of follow-up, after excluding those with related genetic diseases and multiple malformation syndromes and performed late neurological and neuropsychological evaluations and found no significant difference in the verbal intelligence quotient (IQ) (*P* = 0.296), operational IQ (*P* = 0.144), and total score IQ (*P* = 0.065). Hannon et al. (25) also exhibited greater neurodevelopmental delay, language delay, motor delay, learning difficulties, behavioral disorders, neurological deficits, and growth delay after cardiac surgery in the hypothermia group than in the normal temperature group. However, consistent with the results of studies conducted in humans and animals, which have been interpreted as suppression of brain tissue metabolism under HCPB, the present study does not provide obvious evidence that NCPB is superior to HCPB in protecting brain tissues from damage. This study combined with recent high-quality studies shows that NCPB offers advantages of fasting in rewarming during pediatric heart surgery, without causing transient cerebrovascular resistance in the rewarming process and increasing the O_2_-carrying capacity, which may reduce adverse events caused by HCPB and improve the prognosis of children after surgery. This suggests that NCPB has regained attention in pediatric heart surgery, and this gives an extra option for CPB strategies. In other words, the benefits of NCPB may be more than those of HCPB in cases of simple surgery, whereas HCPB may offer more advantages than NCPB in cases of complex surgery. Addition, Corno et al. (24) exhibited that the average cost/patient/day of stay in pediatric intensive care unit (PICU) was lower with NCPB than with HCPB (mean £4,067 ± 3,067 vs. £5,800 ± 4,600, *P* = 0.021). The cost of blood and blood products was also lower in NCPB than in HCPB for the first 24 h after surgery (mean £204 ± 169 vs. mean £306 ± 254, *P* = 0.011). NCPB is similar or superior to HCPB in terms of postoperative renal function, postoperative systemic inflammatory response, and other adverse outcomes. It also reduces the operation time and medical expenses. However, more prospective multicentre RCTs are required to support these results before considering the wide application of NCPB in pediatric heart surgery. Additionally, research must actively focus on investigating adverse events associated with NCPB during or after pediatric heart surgery to fully and objectively analyse the effects of NCPB.

With progression of NCPB research, our knowledge on the optional perfusion technique to ensure proper metabolism during normothermia has increased greatly. Bojan et al. (34) observed that 340-mL min-1 m-2 is probably the lowest suitable? O_2_ required in neonates to maintain aerobic metabolism during NCPB. Zhang et al. (35) exhibited that the lowest suitable? O_2_ during CPB in the infant population undergoing cardiac surgery was 353 ml min^−1^m^−2^, and below this threshold, a high probability of cardiac surgery-associated acute kidney injury (AKI) incidence was noted. Reagor et al. (36) demonstrated that the PS2 group (cardiac index of 3.0 L/min/m^2^, and a nadir hematocrit of 25%) was better than the PS1 group (cardiac index of 2.4 L/min/m^2^, and nadir hematocrit of 28%) in terms of urine output while on CPB (*p* < 0.01) and in all combined postoperative AKI stages (*p* = 0.01), implying that the high cardiac index and oxygen delivery on CPB are associated with low AKI rates. The result may be fulfilled by increasing flow rather than hematocrit, thereby avoiding unnecessary blood transfusions. And it can be found that although avoiding a nadir hematocrit <25% has been well-established, maintaining anything greater than that may not be necessary to achieve adequate oxygen delivery on CPB. The aforementioned studies may be valuable in further improving NCPB outcomes.

The pooled analysis of relevant clinical research data is increasingly being used in evidence-based medicine. If the potential bias is appropriately controlled, high-quality clinical evidence can be provided. And through this meta-analysis and a series of high-quality randomized controlled studies suggest that NCPB treatment has an additional protective effect in pediatric heart surgery and that it releases the medical economic burden.

## Limitations

Although we attempted to control potential bias by using an appropriate methodology, the present study has certain limitations. This study is a meta-analysis of aggregated data and not a separate patient-level study. One of the main limitations of this study is the lack of RCTs. Most of the included trials were retrospective and prospective cohort studies, and the influence of unreported confounding factors cannot be completely ruled out. Moreover, only a part of the outcome indicators included in the study had been reported in the studies, which resulted in low quality and strong heterogeneity in the analysis of relevant data.

## Conclusion

Although the present study observed that NCPB exhibits slightly better results than HCPB in terms of reduced CPB time and serum lactate and creatinine levels, no strong evidence is available to confirm that the adverse events and mortality associated with NCPB are lower than those with HCPB, and the two methods may be similar in terms of safety. More prospective multicentre RCTs are required to explore the advantages of NCPB.

## Data Availability Statement

The original contributions presented in the study are included in the article/Supplementary Material, further inquiries can be directed to the corresponding author/s.

## Author Contributions

TX conceived and designed the research. Y-FM, XC, and Y-LZ developed the search strategies, searched the databases, conducted inspections based on eligibility, and exclusion criteria. LP, XZ, and HL extracted and analyzed quantitative data. Y-XL is the guarantor. All authors contributed to writing, reviewing, or revising this paper.

## Conflict of Interest

The authors declare that the research was conducted in the absence of any commercial or financial relationships that could be construed as a potential conflict of interest.

## Publisher's Note

All claims expressed in this article are solely those of the authors and do not necessarily represent those of their affiliated organizations, or those of the publisher, the editors and the reviewers. Any product that may be evaluated in this article, or claim that may be made by its manufacturer, is not guaranteed or endorsed by the publisher.

## Abbreviations

NCPB: normothermic cardiopulmonary bypass
HCPB: hypothermic cardiopulmonary bypass
CPB: cardiopulmonary bypass
OR: odds ratio
PRISMA: Systematic Reviews and Meta-analysis
WMD: weighted mean difference
ICU: intensive care unit
PICU: pediatric intensive care unit
RCT: randomized controlled trial
DO2: whole-body oxygen delivery
VO2: whole-body oxygen consumption
O2: oxygen
IQ: intelligence quotient
NR: not reported
MD: mean difference
TGA: transposition of the great arteries
VSD: ventricular septal defect
ASD: Atrial septal defect
IAA: interruption of aortic arch
CAA: Coarctation aortic arch
AVSD: atrioventricular septal defect
TOF: tetralogy of Fallot
BSA: body surface area
Kg: kilogram
PC: prospective cohort
RC: retrospective controlled
a: nonpulsatile cardiopulmonary bypass
b: pulsatile cardiopulmonary bypass.
