# Supplementary material for: Safety of Normothermic Cardiopulmonary Bypass in Pediatric Cardiac Surgery: A System Review and Meta-Analysis
Source: Front Pediatr. 2021 Dec 14;9:757551. doi: 10.3389/fped.2021.757551 (PMC8712704; doi:10.3389/fped.2021.757551)
Supplement: Supplementary file 1 [file Data_Sheet_1.docx]

Supplementary Material


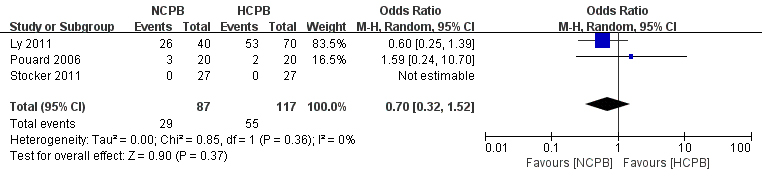


**Figure S1.** Pooled analysis for the comparison of the risk of delayed chest closure.


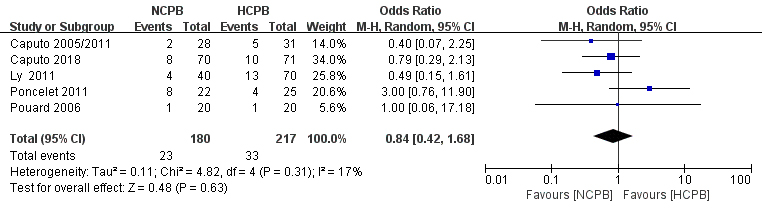


**Figure S2.** Pooled analysis for the comparison of the risk of arrhythmia.


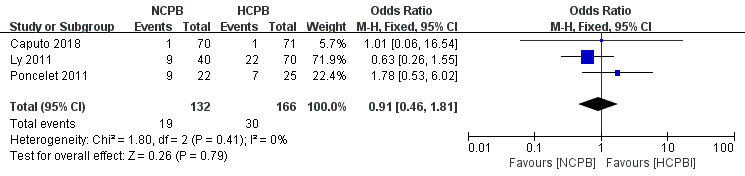


**Figure S3.** Pooled analysis for the comparison of the risk of left ventricular failure.


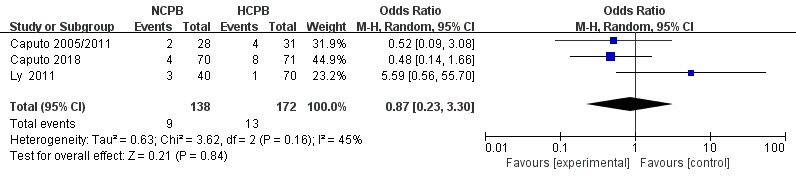


**Figure S4.** Pooled analysis for the comparison of the risk of pulmonary complications.


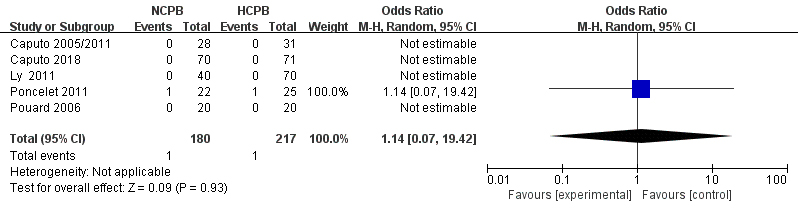


**Figure S5.** Pooled analysis for the comparison of the risk of neurological complications.


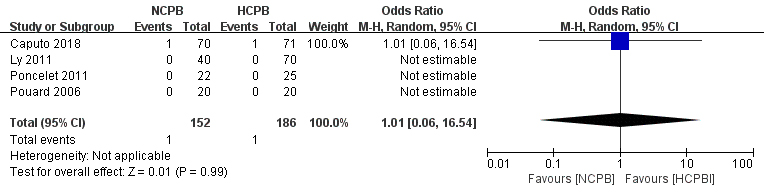


**Figure S6.** Pooled analysis for the comparison of the risk of renal complications.


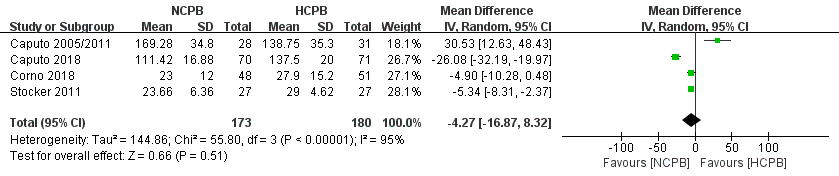


**Figure S7.** Pooled analysis for the comparison of the risk of blood loss.


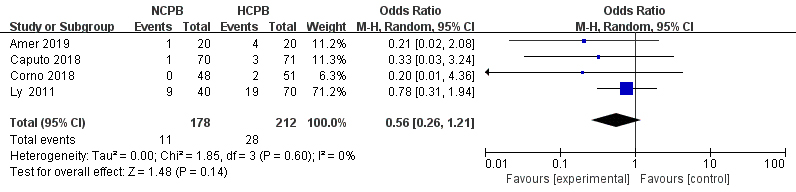


**Figure S8.** Pooled analysis for the comparison of the risk of reoperation.


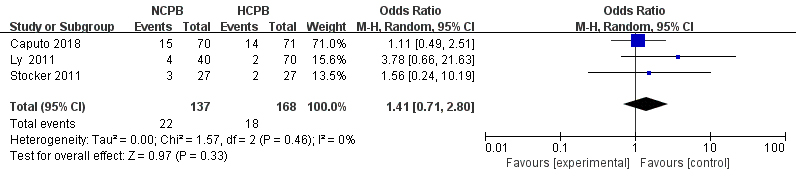


**Figure S9.** Pooled analysis for the comparison of the risk of infective complications.


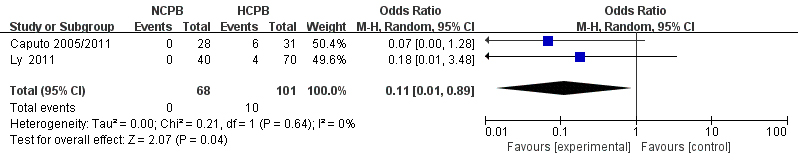


**Figure S10.** Pooled analysis for the comparison of the risk of revision for bleeding.


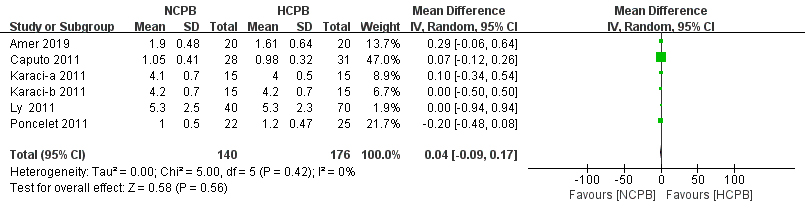


**Figure S11.** Pooled analysis for the comparison of serum lactate levels at baseline.


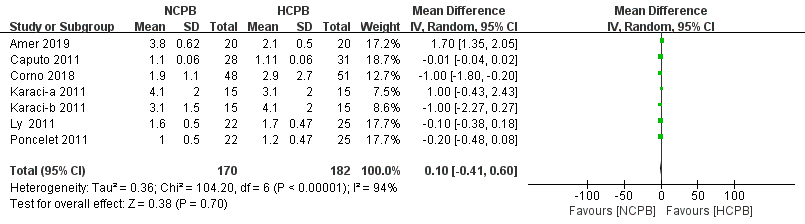


**Figure S12.** Pooled analysis for the comparison of serum lactate levels at the end of CPB.


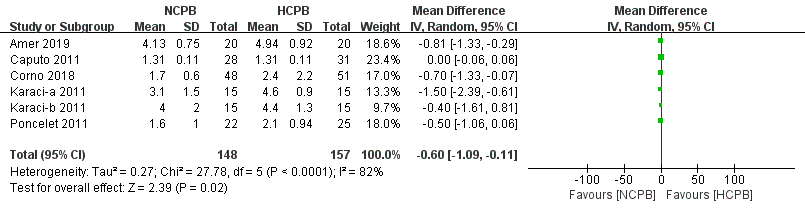


**Figure S13.** Pooled analysis for the comparison of serum lactate levels 2–4 h after CPB.


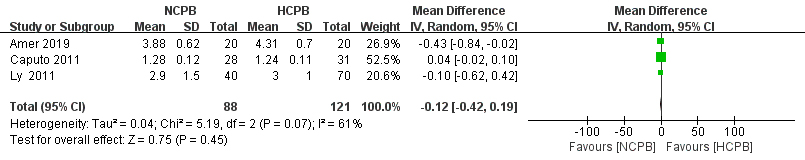


**Figure S14.** Pooled analysis for the comparison of serum lactate levels 6–12 h after CPB.


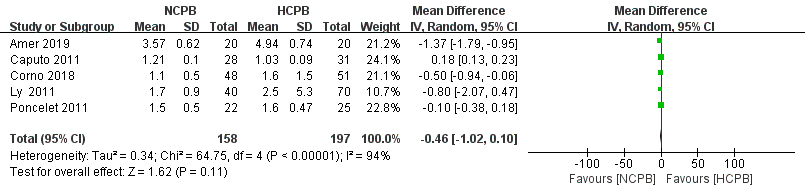


**Figure S15.** Pooled analysis for the comparison of serum lactate levels 12–48 h after CPB.


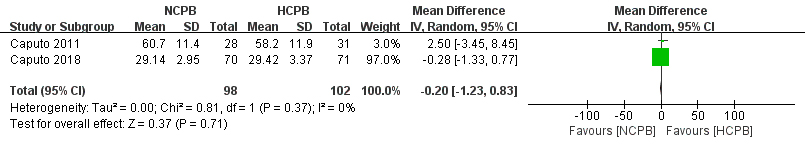


**Figure S16.** Pooled analysis for the comparison of serum creatinemia levels at baseline.


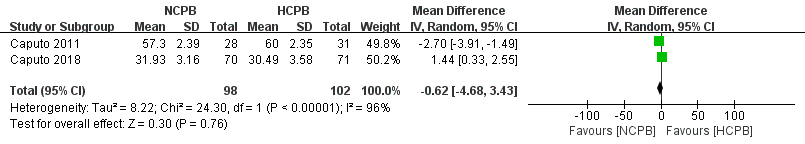


**Figure S17.** Pooled analysis for the comparison of serum creatinemia levels at the end of CPB.


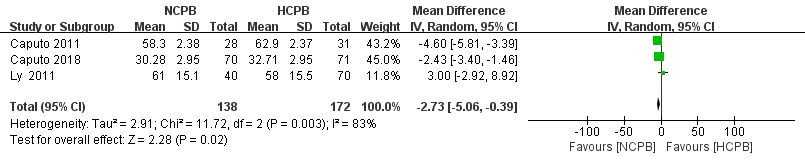


**Figure S18.** Pooled analysis for the comparison of serum creatinemia levels 24 h after CPB.


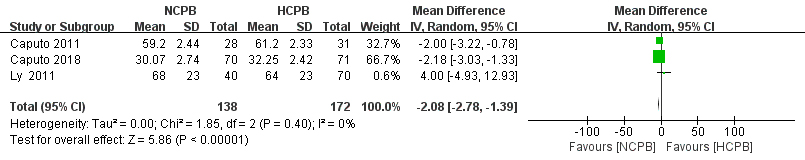


**Figure S19.** Pooled analysis for the comparison of serum creatinemia levels 48 h after CPB.


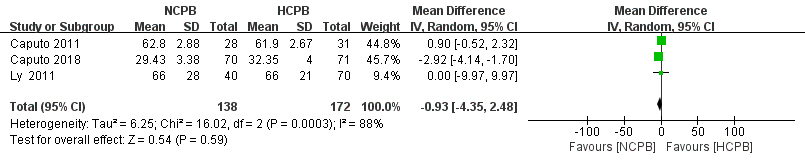


**Figure S20.** Pooled analysis for the comparison of serum creatinemia levels 72 h after CPB.


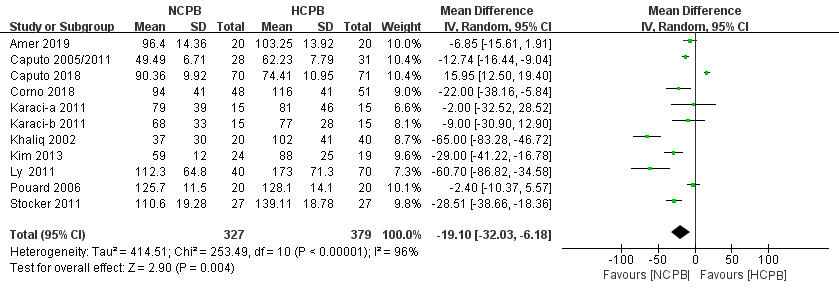


**Figure S21.** Pooled analysis for the comparison of CPB time.


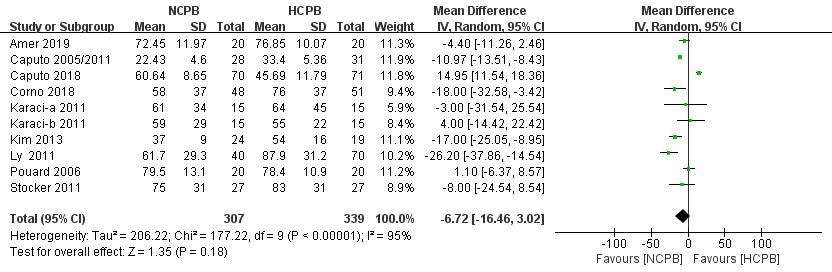


**Figure S22.** Pooled analysis for the comparison of aortic clamping time.


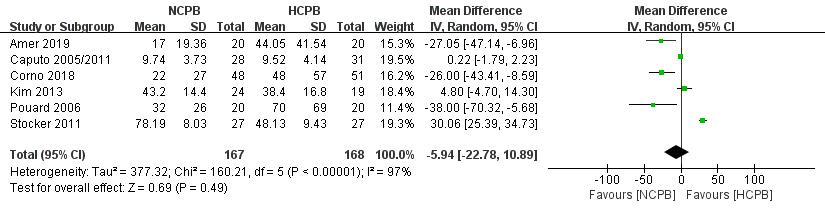


**Figure S23.** Pooled analysis for the comparison of postoperative mechanical ventilation time.

**
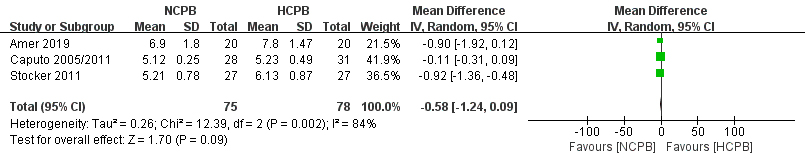
**

**Figure S24.** Pooled analysis for the comparison of time of hospital stay.

**
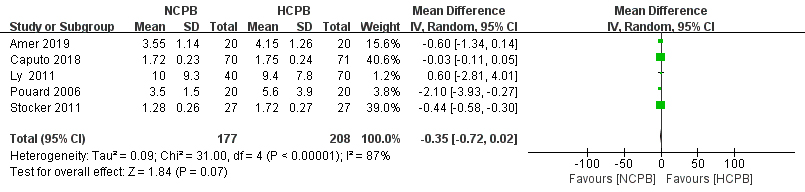
**

**Figure S25.** Pooled analysis for the comparison of time of ICU hospital stay.


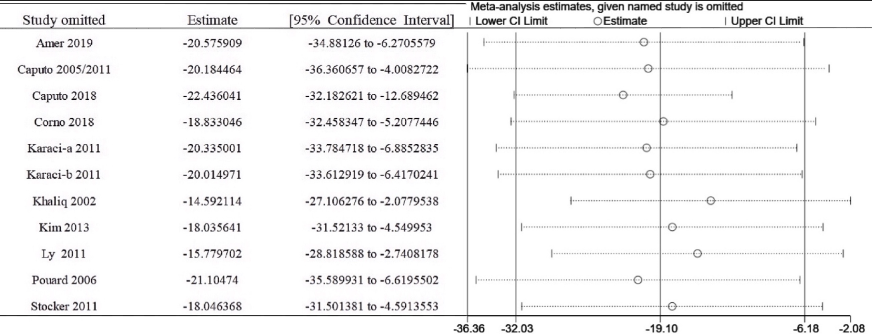


**Figure S26.** Sensitivity analysis of CPB time.

**Table S1.** Data characteristics of postoperative main adverse events.

| Authors, Studies, Year of Publication | delayed chest closure | | arrhythmia | | blood loss/ml | | Left ventricular failure | | Pulmonary Complication | | reopreation | |
| --- | --- | --- | --- | --- | --- | --- | --- | --- | --- | --- | --- | --- |
|  | NCPB group | HCPB group | NCPB group | HCPB group | NCPB group | HCPB group | NCPB group | HCPB group | NCPB group | HCPB group | NCPB group | HCPB group |
| Pouard 2006[22] | 3 | 2 | 1 | 1 | NR | NR | NR | NR | NR | NR | NR | NR |
| Amer 2019[17] | NR | NR | NR | NR | NR | NR | NR | NR | NR | NR | 1 | 4 |
| Caputo 2005[9]/2011[10] | NR | NR | 2 | 5 | 169.28±34.80 | 138.75±35.30 | NR | NR | NR | NR | NR | NR |
| Ly 2011[21] | 26 | 53 | 4 | 13 | NR | NR | 9 | 22 | 3 | 1 | 9 | 19 |
| Poncelet 2011[11] | NR | NR | 8 | 4 | NR | NR | 9 | 7 | NR | NR | NR | NR |
| Corno 2018[18] | NR | NR | NR | NR | 23.0 ± 12.0 | 27.9 ± 15.2 | NR | NR | NR | NR | 0 | 2 |
| caputo 2018[15] | NR | NR | 8 | 10 | 111.42±16.88 | 137.5±20.00 | 1 | 1 | 4 | 8 | 1 | 3 |
| Hannon 2019[19] | NR | NR | NR | NR | NR | NR | NR | NR | NR | NR | NR | NR |
| Kim 2013[20] | NR | NR | NR | NR | NR | NR | NR | NR | NR | NR | NR | NR |
| Stocker 2011[23] | 0 | 0 | NR | NR | 23.66±6.36 | 29±4.62 | NR | NR | NR | NR | NR | NR |

| Neurological event | | Renal complications | | Infective complications | | Respiratory failure | | Revision for bleeding | | death | |
| --- | --- | --- | --- | --- | --- | --- | --- | --- | --- | --- | --- |
| NCPB group | HCPB group | NCPB group | HCPB group | NCPB group | HCPB group | NCPB group | HCPB group | NCPB group | HCPB group | NCPB group | HCPB group |
| 0 | 0 | 0 | 0 | NR | NR | NR | NR | NR | NR | 0 | 0 |
| NR | NR | NR | NR | NR | NR | NR | NR | NR | NR | 0 | 0 |
| NR | NR | NR | NR | NR | NR | NR | NR | 0 | 6 | 0 | 0 |
| 0 | 0 | 0 | 0 | 4 | 2 | NR | NR | 0 | 4 | 4 | 6 |
| 1 | 1 | 0 | 0 | NR | NR | 10 | 4 | NR | NR | 2 | 1 |
| NR | NR | NR | NR | NR | NR | NR | NR | NR | NR | 0 | 0 |
| 0 | 0 | 1 | 1 | 15 | 14 | NR | NR | NR | NR | 0 | 0 |
| 11 | 14 | NR | NR | NR | NR | NR | NR | NR | NR | NR | NR |
| 0 | 0 | NR | NR | NR | NR | NR | NR | NR | NR | NR | NR |
| NR | NR | NR | NR | 3 | 2 | NR | NR | NR | NR | NR | NR |

NCPB, normothermic cardiopulmonary bypass; HCPB, hypothermic cardiopulmonary bypass; NR, no reported.

| **Table S2.** Results of adverse events after surgery pool analysis.   \| Adverse events after surgery \| Results of subgroup pool analysis \| P value \| \| --- \| --- \| --- \| \| delayed chest closure \| OR:0.70;95%CI:0.32-1.52 \| 0.37 \| \| arrhythmia \| OR:0.84;95%CI:0.42-1.68 \| 0.63 \| \| left ventricular failure \| OR:0.91;95%CI:0.46-1.81 \| 0.79 \| \| pulmonary complications \| OR:0.87;95%CI:0.23-3.30 \| 0.84 \| \| neurological complications. \| OR:1.44;95%CI:0.07-19.42 \| 0.93 \| \| renal complications \| OR:1.01;95%CI:0.06-16.54 \| 0.99 \| \| blood loss \| MD:-4.27;95%CI:-16.87-8.32 \| 0.51 \| \| reoperation \| OR:0.56;95%CI:0.26-1.21 \| 0.14 \| \| infective complications \| OR:1.41;95%CI:0.71-2.80 \| 0.33 \| \| revision for bleeding \| OR:1.01;95%CI:0.06-16.54 \| 0.04 \| \| death \| OR:1.39;95%CI:0.43-4.48 \| 0.58 \| \| OR,Odds ratio;MD,Mean Difference;CI,Confidence interval \| \| \|   **Table S3.** Results of serum lactate and serum creatinemia levels pool analysis | |  |  |
| --- | --- | --- | --- | --- | --- | --- | --- | --- | --- | --- | --- | --- | --- | --- | --- | --- | --- | --- | --- | --- | --- | --- | --- | --- | --- | --- | --- | --- | --- | --- | --- | --- | --- | --- | --- | --- | --- | --- | --- | --- | --- | --- |
| Changes in serum lactate and serum creatinemia levels | Results of subgroup pool analysis | | P value |
| serum lactate levels at baseline | MD:0.04;95%CI:-0.09-0.17 | | 0.56 |
| serum lactate levels at the end of CPB | MD:0.10;95%CI:-0.41-0.60 | | 0.7 |
| serum lactate levels 2–4 h after CPB | MD:-0.60;95%CI:-1.09- -0.11 | | 0.02 |
| serum lactate levels 6–12 h after CPB | MD:-0.12;95%CI:-0.42-0.19 | | 0.45 |
| serum lactate levels 12–48 h after CPB | MD:-0.46;95%CI:-1.02-0.10 | | 0.11 |
| serum creatinemia levels at baseline | MD:-0.20;95%CI:-1.23-0.83 | | 0.71 |
| serum creatinemia levels at the end of CPB | MD:-0.62;95%CI:-4.68-3.43 | | 0.76 |
| serum creatinemia levels 24 h after CPB | MD:-2.73;95%CI:-5.06- -0.39 | | 0.02 |
| serum creatinemia levels 48 h after CPB | MD:-2.08;95%CI:-2.78- -1.39 | | ＜0.00001 |
| serum creatinemia levels 72 h after CPB | MD:-0.93;95%CI:-4.35-2.48 | | 0.59 |

OR,Odds ratio;MD,Mean Difference;CI,Confidence interval.

**Table S4.** Serum creatinemia level.

| Authors, Year of Publication | Before CPB (baseline) | | End of CPB | | 24hours after end of CPB | | 48hours after end of CPB | | 72hours after end of CPB | |
| --- | --- | --- | --- | --- | --- | --- | --- | --- | --- | --- |
|  | NCPB  group | HCPB group | NCPB group | HCPB group | NCPB group | HCPB group | NCPB group | HCPB group | NCPB group | HCPB group |
| Caputo 2011[10] | 60.7± 11.4 | 58.2±11.9 | 57.3±2.39 | 60.0±2.35 | 58.3± 2.38 | 62.9± 2.37 | 59.2±2.44 | 61.2±2.33 | 62.8±2.88 | 61.9±2.67 |
| Ly 2011[21] | NR | NR | NR | NR | 61±15.1 | 58±15.5 | 68±23 | 64±23 | 66±28 | 66±21 |
| Caputo 2018[15] | 29.14±2.95 | 29.42±3.37 | 31.93±3.16 | 30.49±3.58 | 30.28±2.95 | 32.71±2.95 | 30.07±2.74 | 32.25±2.42 | 29.43±3.38 | 32.35±4.00 |
| NCPB,normothermic cardiopulmonary bypass;CPB, cardiopulmonary bypass; HCPB,hypothermic cardiopulmonary bypass; NR,no reported; a,nonpulsatile cardiopulmonary bypass; b,pulsatile cardiopulmonary bypass. | | | | | | | | | | |

**Table S5.** Serum lactate level.

| Authors,Year of Publication | Before CPB (baseline) | | End of CPB | | 2-4hours after end of CPB | | 6-12hours after end of CPB | | 12-48hours after end of CPB | |
| --- | --- | --- | --- | --- | --- | --- | --- | --- | --- | --- |
|  | NCPB | HBCP | NCPB | HBCP | NCPB | HBCP | NCPB | HBCP | NCPB | HBCP |
| Amer 2019[17] | 1.9±0.48 | 1.61±0.64 | 3.8±0.62 | 2.1±0.50 | 4.13±0.75 | 4.94±0.92 | 3.88±0.62 | 4.31±0.70 | 3.57±0.62 | 4.94±0.74 |
| Caputo 2011[10] | 1.05±0.41 | 0.98±0.32 | 1.10±0.06 | 1.11±0.06 | 1.31±0.11 | 1.31±0.11 | 1.28±0.12 | 1.24±0.11 | 1.21±0.10 | 1.03±0.09 |
| Ly 2011[21] | 5.3±2.5 | 5.3±2.3 | NR | NR | NR | NR | 2.9±1.5 | 3±1 | 1.7±0.9 | 2.5±5.3 |
| Poncelet 2011[11] | 1.0±0.5 | 1.2±0.47 | 1.6±0.5 | 1.7±0.47 | 1.6±1 | 2.1±0.94 | NR | NR | 1.5±0.5 | 1.6±0.47 |
| Corno 2018[18] | NR | NR | 1.9±1.1 | 2.9±2.7 | 1.7 ± 0.6 | 2.4 ± 2.2 | NR | NR | 1.1 ± 0.5 | 1.6 ± 1.5 |
| Karaci-a 2011[12] | 4.1±0.7 | 4±0.5 | 4.1±2 | 3.1±2 | 3.1±1.5 | 4.6±0.9 | NR | NR | NR | NR |
| Karaci-b 2011[12] | 4.2±0.7 | 4.2±0.7 | 3.1±1.5 | 4.1±2 | 4±2 | 4.4±1.3 | NR | NR | NR | NR |
| NCPB,normothermic cardiopulmonary bypass;CPB, cardiopulmonary bypass; HCPB,hypothermic cardiopulmonary bypass; a,nonpulsatile cardiopulmonary bypass; b,pulsatile cardiopulmonary bypass; NR,no reported. | | | | | | | | | | |

**Table S6.** Intraoperative data, mechanical ventilation time, hospitalisation, and ICU/hospital stay.

|  |
| --- |

|  | Authors,Studies, Year of Publication | | ICU stay/days | | Hospitalisation/days | | Mechanical Ventilation/hours | |  |
| --- | --- | --- | --- | --- | --- | --- | --- | --- | --- |
|  |  |  | NCPB group | HCPB group | NCPB group | HCPB group | NCPB group | HCPB group |  |
|  | Pouard 2006[22] | | 3.5 ± 1.5 | 5.6 ± 3.9 | NR | NR | 32±26 | 70±69 |  |
|  | Amer 2019[17] | | 3.55 ± 1.14 | 4.15 ± 1.26 | 6.90 ± 1.80 | 7.80 ± 1.47 | 17 ± 19.36 | 44.05 ± 41.54 |  |
|  | Caputo 2005[9] | | NR | NR | 5.12 ± 0.25 | 5.23 ± 0.49 | 9.74 ± 3.73 | 9.52 ± 4.14 |  |
|  | Ly 2011[21] | | 10 ± 9.3 | 9.4 ± 7.8 | NR | NR | NR | NR |  |
|  | Poncelet 2011[11] | | 4.38 | 3.87 | NR | NR | NR | NR |  |
|  | Corno 2018[18] | | NR | NR | NR | NR | 22±27 | 48±57 |  |
|  | Caputo 2018[15] | | 1.72±0.23 | 1.75±0.24 | NR | NR | NR | NR |  |
|  | Hannon 2019[19] | | NR | NR | NR | NR | NR | NR |  |
|  | Kim 2013[20] | | NR | NR | NR | NR | 43.2 ± 14.4 | 38.4 ± 16.8 |  |
|  | Stocker 2011[23] | | 1.28 ± 0.26 | 1.72 ± 0.27 | 5.21 ± 0.78 | 6.13 ± 0.87 | 78.19 ± 8.03 | 48.13 ± 9.43 |  |
|  | ICU,Intensive Care Unit;NCPB,normothermic cardiopulmonary bypass; HCPB,hypothermic cardiopulmonary bypass; NR,no reported. | | | | | | | |  |
|  |  |  | |  |  |  |  |  |  |

Table S7. Full statistical table.

| Authors,Studies, Year of Publication | Countries | Operation Period | Study Design | Number of patients | Number of male | |
| --- | --- | --- | --- | --- | --- | --- |
|  |  |  |  | NCBP/HCBP | NCBP  group | HCB group |
| Pouard 2006 | France | October 2000 to October 2001 | PC | 40/(20/20) | NR | NR |
| Amer 2019 | Egypt | October 2017 to February 2019 | RCT | 40/(20/20) | 13 | 12 |
| Caputo 2005/2011 | United Kingdom | November 2002 to November 2004 | RCT | 59/(28/31) | 9 | 16 |
| Ly 2011 | France | 2000 to 2008 | RC | 110/(40/70) | NR | NR |
| Poncelet 2011 | Belgium | May 2004 to September 2005 | RCT | 47/(22/25) | NR | NR |
| Corno 2018 | United States | January 2014 to December 2015 | RC | 99/(48/51) | NR | NR |
| Caputo 2018 | United Kingdom | 2012 to 2014 | RCT | 141/(70/71) | 30 | 31 |
| Hannon 2019 | United Kingdom | January 2014 to December 2015 | RC | 95/(45/50) | 25 | 29 |
| Kim 2013 | Korea | January 2001 to December 2010 | PC | 43/(24/19) | 15 | 11 |
| Stocker 2011 | Australia | March 2003 to July 2005 | RCT | 54/(27/27) | 16 | 16 |
| Karaci-a 2011 | United States | January 19 to March 27, 2011 | PC | 30/(15/15) | 15 | 15 |
| Karaci-b 2011 | United States | January 19 to March 27, 2011 | PC | 30/(15/15) | 15 | 15 |
| Khaliq 2002 | Germany | NR | PC | 49/(20/29) | 20 | 29 |

| delayed chest closure | | arrhythmia | | blood loss/ml | | Left ventricular failure | | Pulmonary Complication | |
| --- | --- | --- | --- | --- | --- | --- | --- | --- | --- |
| NCBP group | HCBP group | NCBP group | HCBP group | NCBP group | HCBP group | NCBP group | HCBP group | NCBP group | HCBP group |
| 3 | 2 | 1 | 1 | NR | NR | NR | NR | NR | NR |
| NR | NR | NR | NR | NR | NR | NR | NR | NR | NR |
| NR | NR | 2 | 5 | 169.28±34.80 | 138.75±35.30 | NR | NR | NR | NR |
| 26 | 53 | 4 | 13 | NR | NR | 9 | 22 | 3 | 1 |
| NR | NR | 8 | 4 | NR | NR | 9 | 7 | NR | NR |
| NR | NR | NR | NR | 23.0 ± 12.0 | 27.9 ± 15.2 | NR | NR | NR | NR |
| NR | NR | 8 | 10 | 111.42±16.88 | 137.5±20.00 | 1 | 1 | 4 | 8 |
| NR | NR | NR | NR | NR | NR | NR | NR | NR | NR |
| NR | NR | NR | NR | NR | NR | NR | NR | NR | NR |
| 0 | 0 | NR | NR | 23.66±6.36 | 29±4.62 | NR | NR | NR | NR |
| NR | NR | NR | NR | NR | NR | NR | NR | NR | NR |
| NR | NR | NR | NR | NR | NR | NR | NR | NR | NR |
| NR | NR | NR | NR | NR | NR | NR | NR | NR | NR |

| reopreation | | Neurological event | | Renal complications | | Infective complications | | Respiratory failure | |
| --- | --- | --- | --- | --- | --- | --- | --- | --- | --- |
| NCBP group | HCBP group | NCBP group | HCBP group | NCBP group | HCBP group | NCBP group | HCBP group | NCBP group | HCBP group |
| NR | NR | 0 | 0 | 0 | 0 | NR | NR | NR | NR |
| 1 | 4 | NR | NR | NR | NR | NR | NR | NR | NR |
| NR | NR | NR | NR | NR | NR | NR | NR | NR | NR |
| 9 | 19 | 0 | 0 | 0 | 0 | 4 | 2 | NR | NR |
| NR | NR | 1 | 1 | 0 | 0 | NR | NR | 10 | 4 |
| 0 | 2 | NR | NR | NR | NR | NR | NR | NR | NR |
| 1 | 3 | 0 | 0 | 1 | 1 | 15 | 14 | NR | NR |
| NR | NR | 11 | 14 | NR | NR | NR | NR | NR | NR |
| NR | NR | 0 | 0 | NR | NR | NR | NR | NR | NR |
| NR | NR | NR | NR | NR | NR | 3 | 2 | NR | NR |
| NR | NR | NR | NR | NR | NR | NR | NR | NR | NR |
| NR | NR | NR | NR | NR | NR | NR | NR | NR | NR |
| NR | NR | NR | NR | NR | NR | NR | NR | NR | NR |

| Revision for bleeding | | death | | ICU stay/days | | Hospitalization/days | | Mechanical Ventilation/hours | |
| --- | --- | --- | --- | --- | --- | --- | --- | --- | --- |
| NCBP group | HCBP group | NCBP group | HCBP group | NCBP group | HCBP group | NCBP group | HCBP group | NCBP group | HCBP group |
| NR | NR | 0 | 0 | 3.5±1.5 | 5.6±3.9 | NR | NR | 32±26 | 70±69 |
| NR | NR | 0 | 0 | 3.55±1.14 | 4.15±1.26 | 6.90±1.80 | 7.80±1.47 | 17±19.36 | 44.05±41.54 |
| 0 | 6 | 0 | 0 | NR | NR | 5.12±0.25 | 5.23±0.49 | 9.74±3.73 | 9.52±4.14 |
| 0 | 4 | 4 | 6 | 10±9.3 | 9.4±7.8 | NR | NR | NR | NR |
| NR | NR | 2 | 1 | 4.38 | 3.87 | NR | NR | NR | NR |
| NR | NR | 0 | 0 | NR | NR | NR | NR | 22±27 | 48±57 |
| NR | NR | 0 | 0 | 1.72±0.23 | 1.75±0.24 | NR | NR | NR | NR |
| NR | NR | NR | NR | NR | NR | NR | NR | NR | NR |
| NR | NR | NR | NR | NR | NR | NR | NR | 43.2±14.4 | 38.4±16.8 |
| NR | NR | NR | NR | 1.28±0.26 | 1.72±0.27 | 5.21±0.78 | 6.13±0.87 | 78.19±8.03 | 48.13±9.43 |
| NR | NR | NR | NR | NR | NR | NR | NR | NR | NR |
| NR | NR | NR | NR | NR | NR | NR | NR | NR | NR |
| NR | NR | NR | NR | NR | NR | NR | NR | NR | NR |

| ICU stay/days | | Hospitalization/days | | Mechanical Ventilation/hours | |
| --- | --- | --- | --- | --- | --- |
| NCBP group | HCBP group | NCBP group | HCBP group | NCBP group | HCBP group |
| 3.5±1.5 | 5.6±3.9 | NR | NR | 32±26 | 70±69 |
| 3.55±1.14 | 4.15±1.26 | 6.90±1.80 | 7.80±1.47 | 17±19.36 | 44.05±41.54 |
| NR | NR | 5.12±0.25 | 5.23±0.49 | 9.74±3.73 | 9.52±4.14 |
| 10±9.3 | 9.4±7.8 | NR | NR | NR | NR |
| 4.38 | 3.87 | NR | NR | NR | NR |
| NR | NR | NR | NR | 22±27 | 48±57 |
| 1.72±0.23 | 1.75±0.24 | NR | NR | NR | NR |
| NR | NR | NR | NR | NR | NR |
| NR | NR | NR | NR | 43.2±14.4 | 38.4±16.8 |
| 1.28±0.26 | 1.72±0.27 | 5.21±0.78 | 6.13±0.87 | 78.19±8.03 | 48.13±9.43 |
| NR | NR | NR | NR | NR | NR |
| NR | NR | NR | NR | NR | NR |
| NR | NR | NR | NR | NR | NR |

| serum lactate level/mmol/L | |
| --- | --- |
| NCBP group | HCBP group |
| NR | NR |
| Before CPB(baseline): 1.9±0.48; During CPB(10min): 2.46±0.5 During CPB(30min): 3.01±0.59 ; 10min after end of CPB: 3.8±0.62 30min after end of CPB: 4.03±0.54; 1hour after end of CPB: 4.13±0.75; after 8hours in the CCU: 3.88±0.62; after 24hours in the CCU: 3.57±0.62 | Before CPB(baseline): 1.61±0.64; During CPB(10min): 2.1±0.50; During CPB(30min): 3.08±0.60; 10min after end of CPB: 3.7±0.73; 30min after end of CPB: 4.39±0.76 ; 1hour after end of CPB: 4.94±0.92 ; after 8hours in the CCU: 4.31±0.70; after 24hours in the CCU: 4.94±0.74 |
| Before CPB(baseline): 1.05±0.41; Crossclamp removal: 0.97±0.06; 0.5hours after crossclamp removal: 1.10±0.06; 2hours after crossclamp removal : 1.31±0.11; 6hours after crossclamp removal: 1.28±0.12; 24hours after crossclamp removal: 1.21±0.10 | Before CPB(baseline): 0.98±0.32; Crossclamp removal: 0.96±0.06; 0.5hours after crossclamp removal: 1.11±0.06; 2hours after crossclamp removal: 1.06±0.10 ;6hours after crossclamp removal: 1.24±0.11 ; 24hours after crossclamp removal: 1.03±0.09 |
| Before CPB(baseline): 5.3±2.5; 12hours after end of CPB: 2.9±1.5;  24hours after end of CPB: 1.7±0.9 | Before CPB(baseline): 5.3±2.3 ; 12hours after end of CPB: 3±1; 24hours after end of CPB: 2.5±5.3 |
| Before CPB(baseline): 1.0±0.5; 0.05hours after coronary reperfusion: 2.3±1; 2hours after coronary reperfusion: 1.6±0.5; 6hours after coronary reperfusion: 1.6±1; 12hours after coronary reperfusion: 1.5±0.5 | Before CPB(baseline): 1.2±0.47; 0.05hours after coronary reperfusion: 2.4±0.94; 2hours after coronary reperfusion: 1.7±0.47; 6hours after coronary reperfusion: 2.1±0.94; 12hours after coronary reperfusion: 1.6±0.47 |
| PICU arrival: 1.9±1.1; 4hours after PICU arrival: 1.7 ± 0.6; 24hours after PICU arrival: 1.1 ± 0.5 | PICU arrival: 2.9±2.7; 4hours after PICU arrival: 2.4 ± 2.2 ; 24hours after PICU arrival: 1.6 ± 1.5 |
| NR | NR |
| NR | NR |
| NR | NR |
| NR | NR |
| Before CPB(baseline): 4.1±0.7; During CPB(2-5min): 3.3±1.5; 20 min after during crossclamp: 3.4±2; 60 min during crossclamp: 4.1±2; End of CPB: 4.1±2 ; 2hours after end of CPB: 3.1±1.5 | Before CPB(baseline): 4±0.5; During CPB(2-5min): 3.3±1.2; 20 min during crossclamp: 4.4±2; 60 min during crossclamp: 3.1±2 ; End of CPB: 3.1±2 ; 2hours after end of CPB: 4.6±0.9 |
| Before CPB(baseline): 4.2±0.7; During CPB(2-5min): 3.1±1.2; 20 min during crossclamp: 3.1±1.5; 60 min during crossclamp: 3.1±1.5; End of CPB: 3.1±1.5; 2hours after end of CPB: 4±2 | Before CPB(baseline): 4.2±0.7; During CPB(2-5min): 3.3±0.7 ; 20 min during crossclamp: 3.1±2 60 min during crossclampp: 4.1±2; End of CPB: 4.1±2; 2hours after end of CPB: 4.4±1.3 |
| NR | NR |

| serum creatinemia level/mmol/L | |
| --- | --- |
| NCBP group | HCBP group |
| NR | NR |
| NR | NR |
| Before CPB(baseline): 60.7± 11.4; End of CPB: 57.3±2.39; 24hours after end of CPB: 58.3± 2.38; 48hours after end of CPB: 59.2±2.44 ; 72hours after end of CPB: 62.8±2.88 | Before CPB(baseline): 58.2±11.9; End of CPB: 60.0±2.35 ; 24hours after end of CPB: 62.9± 2.37; 48hours after end of CPB: 61.2±2.33; 72hours after end of CPB: 61.9±2.67 |
| 24hours after end of CPB: 61±15.1; 48hours after end of CPB: 68±23; 72hours after end of CPB: 66±28 | 24hours after end of CPB: 58±15.5 48hours after end of CPB: 64±23 72hours after end of CPB: 66±21 |
| NR | NR |
| NR | NR |
| Pre-operative(baseline): 29.14±2.95; Operation end: 31.93±3.16; 24hours after operation end: 30.28±2.95; 48hours after operation end: 30.07±2.74; 72hours after operation end: 29.43±3.38 | Pre-operative(baseline): 29.42±3.37; Operation end: 30.49±3.58 ; 24hours after operation end: 32.71±2.95; 48hours after operation end: 32.25±2.42; 72hours after operation end: 32.35±4.00 |
| NR | NR |
| NR | NR |
| NR | NR |
| NR | NR |
| NR | NR |
| NR | NR |
