# Supplementary material for: Safety of Normothermic Cardiopulmonary Bypass in Pediatric Cardiac Surgery: A System Review and Meta-Analysis
Source: Front Pediatr. 2021 Dec 14;9:757551. doi: 10.3389/fped.2021.757551 (PMC8712704; doi:10.3389/fped.2021.757551)
Supplement: Supplementary file 2 [file Data_Sheet_2.pdf]

Additional file 1. Search strategy for each database

Database	Search strategy
PubMed	((((((normal temperature) OR (normal thermic)) OR (normothermic)) OR (normothermia)) AND (((((((((Surgery, Thoracic) OR (Surgery, Cardiac)) OR (Surgery, Heart)) OR (Heart Surgery)) OR (Cardiac Surgery)) OR ("Thoracic Surgery"[Mesh])) OR ((((((cardiac surgery) OR (cardiothoracic surgery)) OR (heart surgery)) OR (congenital heart surgery)) OR (Pediatric Cardiology)) OR ((((((((((((Procedure, Cardiac Surgical) OR (Procedures, Cardiac Surgical)) OR (Surgical Procedure, Cardiac)) OR (Surgical Procedures, Cardiac)) OR (Surgical Procedures, Heart)) OR (Cardiac Surgical Procedure)) OR (Heart Surgical Procedures)) OR (Procedure, Heart Surgical)) OR (Procedures, Heart Surgical)) OR (Surgical Procedure, Heart)) OR (Heart Surgical Procedure)) OR ("Cardiac Surgical Procedures"[Mesh])))) OR (((((((((Heart-Lung Bypass) OR (Bypass, Heart-Lung)) OR (Bypasses, Heart-Lung)) OR (Heart Lung Bypass)) OR (Heart-Lung Bypasses)) OR (Bypass, Cardiopulmonary)) OR (Bypasses, Cardiopulmonary)) OR (Cardiopulmonary Bypasses)) OR ("Cardiopulmonary Bypass"[Mesh]))) OR ((((((Circulation, Extracorporeal) OR (Circulations, Extracorporeal)) OR (Extracorporeal Circulations)) OR (CPB)) OR (bypass)) OR ("Extracorporeal Circulation"[Mesh])))) AND ((((((((((((Infants, Newborn) OR (Newborn Infant)) OR (Newborn Infants)) OR (Newborns)) OR (Newborn)) OR (Neonate)) OR (Neonates)) OR ("Infant, Newborn"[Mesh])) OR ("Pediatrics"[Mesh])) OR ((children) OR ("Child"[Mesh]))) OR ((Infants) OR ("Infant"[Mesh]))) OR (pedia)))
Embase	('pediatric'/exp OR ('child'/exp OR 'children':ab,ti) OR ('infant'/exp OR 'infants':ab,ti) OR pedia OR ('newborn'/exp OR 'infants, newborn':ab,ti OR 'newborn infant':ab,ti OR 'newborn infants':ab,ti OR 'newborns':ab,ti OR 'neonate':ab,ti OR 'neonates':ab,ti OR 'infant, newborn':ab,ti)) AND (('thorax surgery'/exp OR 'surgery, thoracic':ab,ti OR 'surgery, cardiac':ab,ti OR 'surgery, heart':ab,ti OR 'heart surgery':ab,ti OR 'cardiac surgery':ab,ti OR 'thoracic surgery':ab,ti) OR ('heart surgery'/exp OR 'procedure, cardiac surgical':ab,ti OR 'procedures, cardiac surgical':ab,ti OR 'surgical procedure, cardiac':ab,ti OR 'surgical procedures, cardiac':ab,ti OR 'surgical procedures, heart':ab,ti OR 'cardiac surgical procedure':ab,ti OR 'heart surgical procedures':ab,ti OR 'procedure, heart surgical':ab,ti OR 'procedures, heart surgical':ab,ti OR 'surgical procedure, heart':ab,ti OR 'heart surgical procedure':ab,ti OR 'cardiac surgical procedures':ab,ti OR 'cardiac surgery':ab,ti OR 'cardiothoracic surgery':ab,ti OR 'heart surgery':ab,ti OR 'congenital heart surgery':ab,ti OR 'pediatric cardiology':ab,ti) OR ('cardiopulmonary bypass'/exp OR 'heart-lung bypass':ab,ti OR 'bypass, heart-lung':ab,ti OR 'bypasses, heart-lung':ab,ti OR 'heart lung bypass':ab,ti OR 'heart-lung bypasses':ab,ti OR 'bypass, cardiopulmonary':ab,ti OR 'bypasses, cardiopulmonary':ab,ti OR 'cardiopulmonary bypasses':ab,ti) OR ('extracorporeal circulation'/exp OR 'circulation, extracorporeal':ab,ti OR 'circulations, extracorporeal':ab,ti OR 'extracorporeal circulations':ab,ti OR 'cpb':ab,ti OR 'bypass':ab,ti)) AND ('normal temperature':ab,ti OR 'normal thermic':ab,ti OR 'normothermic':ab,ti OR 'normothermia':ab,ti))

Cochrane Library	1. MeSH descriptor: [undefined] explode all trees<br>2. MeSH descriptor: [Child] explode all trees<br>3. (children):ti,ab,kw (Word variations have been searched)<br>4. #3 OR #2<br>5. MeSH descriptor: [Infant] explode all trees<br>6. (Infants):ti,ab,kw OR (pedia):ti,ab,kw (Word variations have been searched)<br>7. #5 OR #6<br>8. MeSH descriptor: [Infant, Newborn] explode all trees<br>9. (Infants, Newborn):ti,ab,kw OR (Newborn Infant):ti,ab,kw OR (Newborn Infants):ti,ab,kw OR (Newborns):ti,ab,kw OR (Newborn):ti,ab,kw OR (Neonate):ti,ab,kw OR (Neonates):ti,ab,kw<br>10. #8 OR #9<br>11. #1 OR #4 OR #7 OR #10<br>12. MeSH descriptor: [Thoracic Surgery] explode all trees<br>13. (Surgery, Thoracic):ti,ab,kw OR (Surgery, Cardiac):ti,ab,kw OR (Surgery, Heart):ti,ab,kw OR (Heart Surgery):ti,ab,kw OR (Cardiac Surgery):ti,ab,kw<br>14. #12 OR 13<br>15. MeSH descriptor: [Cardiac Surgical Procedures] explode all trees<br>16. (Procedure, Cardiac Surgical):ti,ab,kw OR (Procedures, Cardiac Surgical):ti,ab,kw OR (Surgical Procedure, Cardiac):ti,ab,kw OR (Surgical Procedures, Cardiac):ti,ab,kw OR (Surgical Procedures, Heart):ti,ab,kw OR (Cardiac Surgical Procedure):ti,ab,kw OR (Heart Surgical Procedures):ti,ab,kw OR (Procedure, Heart Surgical):ti,ab,kw OR (Procedures, Heart Surgical):ti,ab,kw OR (Surgical Procedure, Heart):ti,ab,kw OR (Heart Surgical Procedure):ti,ab,kw OR (cardiac surgery):ti,ab,kw OR (cardiothoracic surgery):ti,ab,kw OR (heart surgery):ti,ab,kw OR (congenital heart surgery):ti,ab,kw OR (Pediatric Cardiology):ti,ab,kw<br>17. #15 OR #16<br>18. MeSH descriptor: [Cardiopulmonary Bypass] explode all trees<br>19. (Heart-Lung Bypass):ti,ab,kw OR (Bypass, Heart-Lung):ti,ab,kw OR (Bypasses, Heart-Lung):ti,ab,kw OR (Heart Lung Bypass):ti,ab,kw OR (Heart-Lung Bypasses):ti,ab,kw OR (Bypass, Cardiopulmonary):ti,ab,kw OR (Bypasses, Cardiopulmonary):ti,ab,kw OR (Cardiopulmonary

	Bypasses):ti,ab,kw<br>20. #18 OR #19<br>21. MeSH descriptor: [Extracorporeal Circulation] explode all trees<br>22. (Circulation, Extracorporeal):ti,ab,kw OR (Circulations, Extracorporeal):ti,ab,kw OR (Extracorporeal Circulations):ti,ab,kw OR (CPB):ti,ab,kw OR (bypass):ti,ab,kw<br>23. #21 OR #22<br>24. #14 OR #17 OR #20 OR #23<br>25. (normal temperature):ti,ab,kw OR (normal thermic):ti,ab,kw OR (normothermic):ti,ab,kw OR (normothermia):ti,ab,kw 4646<br>26. #11 AND #24 AND #25
Clinical Trials.gov	**Condition or disease:** cardiac surgery OR cardiothoracic surgery OR heart surgery OR congenital heart surgery OR Pediatric Cardiology OR cardiopulmonary bypass OR CPB<br>**Other terms:** normal temperature OR normal thermic OR normothermic OR normothermia<br>**Study type:** All studies<br>**Study Results:** All studies
